# Multi-locus analysis supports the taxonomic validity of *Arborophila gingica guangxiensis* Fang Zhou & Aiwu Jiang, 2008

**DOI:** 10.3897/zookeys.555.6814

**Published:** 2016-01-20

**Authors:** De Chen, Qiong Liu, Jiang Chang, Aiwu Jiang, Fang Zhou, Yanyun Zhang, Zhengwang Zhang

**Affiliations:** 1 MOE Key Laboratory for Biodiversity Science and Ecological Engineering, College of Life Sciences, Beijing Normal University, Beijing 100875, China; 2 State Key Laboratory of Environmental Criteria and Risk Assessment, Chinese Research Academy of Environmental Sciences, Beijing 100012, China; 3 College of Forestry, Guangxi University, Nanning 530004, Guangxi, China; 4 College of Animal Science and Technology, Guangxi University, Nanning 530004, Guangxi, China

**Keywords:** Conservation unit, evolutionary distinctiveness, hill partridge, mitochondrial DNA, nuclear introns, monophyletic groups

## Abstract

The taxonomic status of subspecies has long been debated, especially in conservation biology. Some proposed subspecies must be evolutionarily distinct to be considered conservation units. White-necklaced Partridge (*Arborophila gingica*) comprises two subspecies, *Arborophila gingica gingica* and *Arborophila gingica guangxiensis*. *Arborophila gingica guangxiensis*, restricted to three isolated small areas in Guangxi, China, with limited population sizes, is a newly discovered subspecies based on recently identified geographic and phenotypic differences between *Arborophila gingica gingica*; however, evidence is lacking that can effectively identify whether the subspecies is evolutionarily distinct. Here, three mitochondrial DNA segments and four nuclear introns were used to test whether the two subspecies are reciprocally monophyletic, which has been proposed as an objective method to evaluate evolutionary distinctiveness. The results indicate that the two subspecies are genetically divergent and form reciprocal monophyletic groups. Therefore, this study further supports the taxonomic validity and distinctiveness of *Arborophila gingica guangxiensis* and suggests that this subspecies be considered as a conservation unit.

## Introduction

The taxonomic status of subspecies has long been debated (Wilson and Brown 1953; Mayr 1982), especially in resource-limited conservation biology (Zink 2004; Phillimore and Owens 2006). Zink (2004) proposed that subspecies must be evolutionarily distinct to be considered conservation units. However, a recent global analysis showed that only 36% of traditional avian subspecies can be distinguishable by mitochondrial DNA (mtDNA) (Phillimore and Owens 2006). Therefore, assessing the validity of subspecies before proposing conservation efforts may be a practical way to effectively protect biodiversity with limited resources.

**Figure 1.**
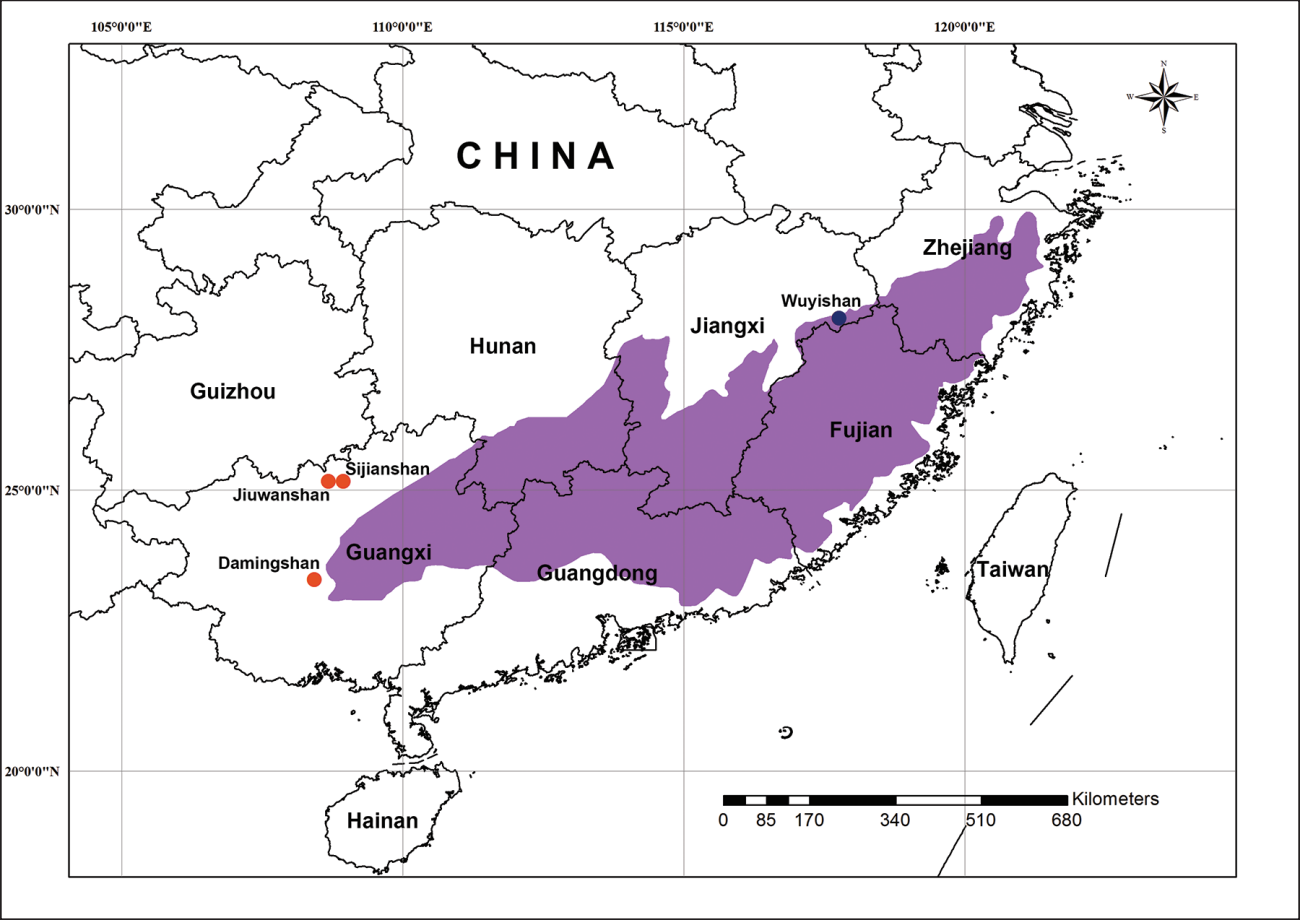
Map of southeast China showing the distribution area of *Arborophila gingica*. The purple area represents the distribution of the nominate subspecies *Arborophila gingica gingica* according to del Hoyo and Collar (2014), with the dark blue dot indicating the sampling site in Wuyishan Mountain. The orange dots represent the three isolated populations of *Arborophila gingica guangxiensis* (Zhou and Jiang 2008), with the sampling site in Jiuwanshan Mountain.

The near threatened (NT) White-necklaced Partridge (*Arborophila gingica*) (BirdLife International 2012), also known as the Collared or Rickett’s hill partridge, is a small partridge endemic to the southern Chinese forests of Hunan, Jiangxi, Zhejiang, Fujian, Guangdong and Guangxi (Fig. 1, Cheng 1978; Zheng 2011). Although the distribution area appears extensive, populations are severely fragmented and continuously declining (Zhou 1996; BirdLife International 2012), except for the Fujian population (He et al. 2007). This species was believed to be monotypic (Johnsgard 1988; Madge and McGowan 2002) until Zhou and Jiang (2008) discovered that the populations in north and central Guangxi differed from other southeastern populations in the coloration of the forehead, which is chestnut instead of white. Zhou and Jiang (2008) described these populations as a new subspecies, *Arborophila gingica guangxiensis*, a proposal subsequently accepted by several avian checklists (Zheng 2011; Dickinson and Remsen 2013; del Hoyo and Collar 2014; Gill and Donsker 2015). However, except for its diagnostic forehead coloration, no other plumage differences are known, and body weight, body length, wing, culmen, tarsus and tail show no obvious differences between the two subspecies (Zhou and Jiang 2008). *Arborophila gingica guangxiensis* is only found in three isolated small areas of north (Jiuwanshan Mountain and Sijianshan Mountain) and central (Damingshan Mountain) Guangxi (Fig. 1, Zhou and Jiang 2008). The estimated population is about 600 to 1000 individuals, thus undoubtedly *Arborophila gingica guangxiensis* suffers more threats than *Arborophila gingica gingica* (Taylor 2011). However, the subspecies status of *Arborophila gingica guangxiensis* is solely dependent on geographic and phenotypic differences, lacking molecular evidence that can effectively identify whether this isolated subspecies is evolutionarily distinct and can be considered a conservation unit (Zink 2004). Indeed, some operational criteria for subspecies recognition require that subspecies are both phenotypically distinct and correlate with evolutionary independence according to population genetic structure (Braby et al. 2012).

Recently, molecular systematics has become one of the most vigorous disciplines to assist in avian taxonomy (Fjeldså 2013). Specifically, mtDNA has been extensively used at various taxonomic levels (Zink and Barrowclough 2008). Zink (2004) advocated that subspecies should be reciprocally monophyletic in mtDNA gene trees to document the evolutionary distinctiveness of subspecies. However, studies that are based solely on mtDNA have been debated because population differentiation relies on the accumulated signals from many genes and mtDNA only represents a single locus (Rubinoff and Holland 2005; Edwards and Bensch 2009). Therefore, a reasonable strategy for phylogenetic analysis is to combine mtDNA with nuclear DNA (nuDNA) sequences (Corl and Ellegren 2013).

Here, three mtDNA segments and four nuclear introns of White-necklaced Partridge were combined to conduct a series of phylogenetic analyses and test whether *Arborophila gingica guangxiensis* and *Arborophila gingica gingica* form reciprocally monophyletic groups. Furthermore, times of divergence within *Arborophila gingica*, between *Arborophila gingica* and its closest relative, were investigated, and attempts to identify possible drivers of the diversification process were made.

## Methods

### Sampling, DNA extraction, PCR and sequencing

Seven individuals of *Arborophila gingica guangxiensis* were sampled from Jiuwanshan National Nature Reserve, Guangxi, and three individuals of *Arborophila gingica gingica* from Wuyishan National Nature Reserve, Jiangxi (Fig. 1). Previous studies indicated that the sister species of *Arborophila gingica* was *Arborophila rufogularis* (Wang et al. 2013); therefore, we used one individual of *Arborophila rufogularis* from Tongbiguan National Nature Reserve in Yunnan as an outgroup. All samples were taken from live birds (blood or feather). Permissions for blood or feather sampling were granted by the regional forestry departments. Total DNA was extracted using a TIANamp Blood Genomic DNA Extraction Kit (TIANGEN BIOTECH CO, BEIJING, CHINA).

We amplified three mtDNA segments, cytochrome oxidase subunit 1 (COI), cytochrome *b*
(CYTB) and NADH dehydrogenase subunit 2 (ND2); and four nuclear introns, aldolase b intron 6 (ALDOB), fibrinogen intron 5 (FGB), glyceraldehyde 3-phosphate dehydrogenase intron 11 (G3PDH) and ovomucoid intron G (OVOG) using the primers listed in Suppl. material 1. Both strands of each PCR product were sequenced by BGI-BEIJING. The sequences were visually proofread to the original chromatograms and were also checked against published DNA sequences. Each sequence was then assembled using MEGA v6 (Tamura et al. 2013). Then, we aligned the sequences using MUSCLE (Edgar 2004) implemented in MEGA v6 (Tamura et al. 2013) to obtain seven partitions, all sequences obtained from this study were submitted to GenBank (KU057820–KU057877). Each nuclear partition was then phased (Stephens and Donnelly 2003) in DNASP v5.10 (Librado and Rozas 2009) to resolve the haplotypes of diploid nuclear sequences. Finally, we assembled the seven partitions into a complete matrix, an mtDNA matrix and a nuDNA matrix.

### Phylogenetic analysis

The best-ﬁtting nucleotide substitution model for each partition was selected using the Akaike Information Criterion with JMODELTEST v2.1.7 (Darriba et al. 2012). The mean genetic distances between and within subspecies were calculated in MEGA v6 (Tamura et al. 2013) using the Kimura two-parameter (K2P) model (with *Arborophila rufogularis* removed); and standard error estimates were obtained by a bootstrap procedure (1000 replicates). Partitioned maximum likelihood (ML) analyses were conducted in GARLI v2.0 (Bazinet et al. 2014) using the best-fitting nucleotide substitution model for each partition. The subtree pruning and regrafting tree-searching method was used, and bootstrap values (BS) were calculated with 1,000 replicates. Partitioned Bayesian Inference (BI) was performed in BEAST v1.8.0 (Drummond and Rambaut 2007) with the best-fitting nucleotide substitution model for each partition (similar to Divergence time estimates, see below for details).

### Divergence time estimates

First, we performed molecular clock tests in MEGA v6 (Tamura et al. 2013). The results showed that each partition was clock-like. Therefore we used the strict clock model for each partition. It is believed that a species tree analysis using combined mtDNA, Z-linked (ALDOB) and autosomal (FGB, G3PDH and OVOG) loci can substantially improve the resolution of the tree (Corl and Ellegren 2013). Therefore, we performed a species tree analysis using the complete matrix in *BEAST (Heled and Drummond 2010) implemented in BEAST v1.8.0 (Drummond and Rambaut 2007), with a fixed molecular rate of 2.38% for CYTB (average molecular rate for Galliform birds, Weir and Schluter (2008)) to estimate the molecular rates of the other loci. The ESS value was verified to be greater than 200 in TRACER v1.5 (Rambaut and Drummond 2009) to confirm that the chains had reached apparent stationarity. The ﬁnal analysis was run for 100 million generations with trees sampled every 1,000 generations. TreeAnnotator v1.8.0 was then used to discard the ﬁrst 20% of trees and to generate the consensus tree with Bayesian posterior probability.

## Results

The complete matrix was 4750 base pairs (bp) in length, including 2861 bp of mtDNA sequence data, and 1889 bp of nuclear intron sequence data. Exclude outgroup, there were 18 variable and 13 informative sites in mtDNA, and 24 variable and 19 informative sites in nuDNA (after phasing). The genetic distance between the two subspecies was higher in mtDNA (0.0038) than in nuDNA (0.0028), and in nuclear introns the genetic distance within subspecies partially overlapped with that between subspecies (Table 1) due to some shared haplotypes (data not shown).

**Figure 2.**
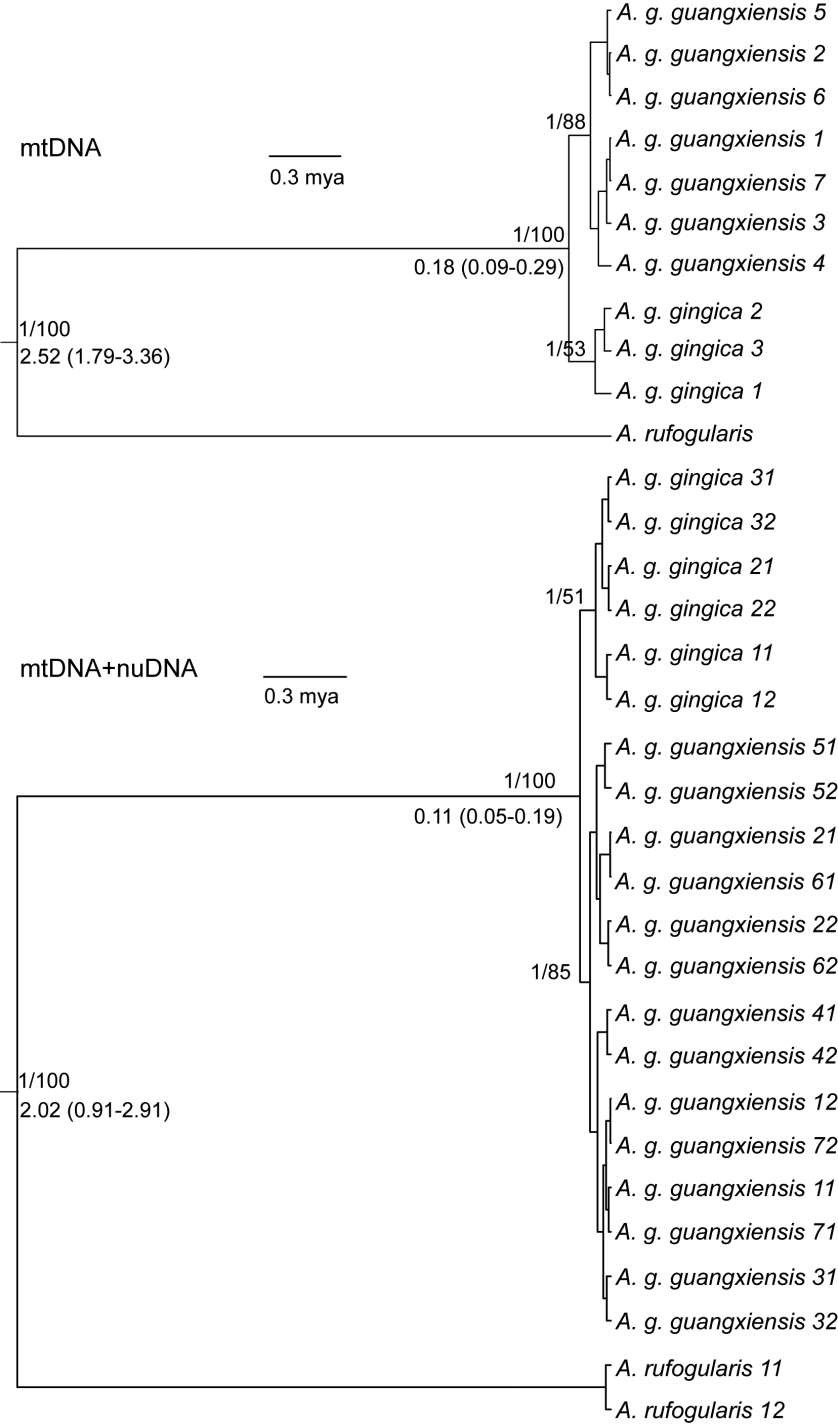
Phylogenetic consensus trees from the mtDNA matrix and complete data matrix. Node values above the branches represented the BI posterior probability and ML bootstrap support. Values below the branches represent the divergence times (median) and 95% highest posterior density (HPD) between lineage groups, note that the divergence times in the multi-locus tree were estimated by species tree analysis. The last number in tip labels in the multi-locus tree represent the two haplotypes phased from diploid nuclear sequences.

Phylogenetic analyses of the complete matrix and mtDNA matrix showed that *Arborophila gingica guangxiensis* and *Arborophila gingica gingica* formed monophyletic groups, with relatively high support (Fig. 2). However, analyses of the nuDNA matrix and separate analyses of each of the nuclear introns failed to recover the monophyletic relationships between *Arborophila gingica guangxiensis* and *Arborophila gingica gingica* (Suppl. material 2), and the support values are extremely low (data not shown).

Divergence time estimates from the species tree showed that the two subspecies *Arborophila gingica guangxiensis* and *Arborophila gingica gingica* diverged approximately 0.11 (0.05–0.19) mya (million years ago), whereas the divergence between *Arborophila gingica* and *Arborophila rufogularis* occurred 2.02 (0.91–2.91) mya (Fig. 2).

**Table 1.** Mean genetic distances (K2P) between and within subspecies.

Mean distance	mtDNA	ALDOB	FGB	G3PDH	OVOG	nuDNA
Within *guangxiensis*	0.0013 ±0.0005	0.0017 ±0.0009	0.0032 ±0.0012	0.0037 ±0.0017	0.0032 ±0.0015	0.0029 ±0.0007
Within *gingica*	0.0012 ±0.0005	0.0013 ±0.0012	0.0021 ±0.0012	0.0009 ±0.0009	0.0019 ±0.0013	0.0016 ±0.0007
Between subspecies	0.0038 ±0.0009	0.0017 ±0.0011	0.0033 ±0.0012	0.0024 ±0.0011	0.0038 ±0.0018	0.0028 ±0.0007

Standard errors are shown after the symbol “±”

## Discussion

This study documents genetic differentiation between *Arborophila gingica guangxiensis* and *Arborophila gingica gingica*. The phylogenetic analyses based on mtDNA indicate that *Arborophila gingica guangxiensis* and *Arborophila gingica gingica* form reciprocal monophyletic groups (Fig. 2), which meets the criterion that subspecies should be monophyletic in mtDNA to demonstrate evolutionary distinctiveness (Zink 2004). Monophyly was also supported by the multi-locus tree (Fig. 2).

However, although the *Arborophila gingica guangxiensis* clade received high support in the mtDNA tree (Fig. 2), nuDNA trees failed to recover the two subspecies as monophyletic groups (Suppl. material 2). This difference might be explained by the longer coalescence time of nuDNA due to its larger effective population size than mtDNA (Moore 1995), so that in recently diverged taxa lineage sorting would be complete for mtDNA but not yet for nuDNA (Zink and Barrowclough 2008). Between *Arborophila gingica guangxiensis* and *Arborophila gingica gingica*, mtDNA haplotypes were fully sorted whereas both taxa had a few shared nuDNA haplotypes. Furthermore, the combined mtDNA and nuDNA tree showed that *Arborophila gingica guangxiensis* and *Arborophila gingica gingica* formed reciprocally monophyletic groups (Fig. 2). Although the monophyly was mainly resolved by mtDNA (Zink and Barrowclough 2008), our results indicate that the two subspecies already exhibit recognizable divergences in nuDNA haplotype frequency, although the divergence was not complete.

In general, molecular phylogenetic study often reveals non-monophyly of avian subspecies (Zink 2004, Phillimore and Owens 2006), which may be due to incorrect taxonomy or rapid divergence. Among Chinese birds, non-monophyly has been documented in some of the subspecies of *Lophura nycthemera* (Dong et al. 2013), *Charadrius alexandrinus* (Rheindt et al. 2011) and *Garrulax chinensis* (Wu et al. 2012), and all subspecies of *Motacilla alba* (Li et al. 2015) and *Leucosticte brandti* (Sangster et al. 2015). Thus, the congruent divergence of morphological and molecular markers in *Arborophila gingica* contrasts with the divergence patterns observed in several other avian species. However, many tropical and subtropical subspecies have been shown to be monophyletic, and sometimes highly divergent, underscoring the necessity of phylogeographic study for taxonomy within species (e.g. Song et al. 2009; Irestedt et al. 2013).

The divergence between *Arborophila gingica* and *Arborophila rufogularis* in southwest China (Chen et al. 2015) occurred approximately 2.02 mya when there was a major uplift of the Yunnan-Guizhou Plateau during the Plio-Pleistocene boundary (1.8–2.6 mya) (Zhang and Fang 2012). The uplift may have promoted geographical isolation in many species during this period (Qu et al. 2015), including *Stachyridopsis ruficeps* (Liu et al. 2012) and *Aegithalos concinnus* (Dai et al. 2011). The dramatic climatic cooling during the Plio-Pleistocene boundary may have resulted in altitudinal shifts in montane species (Hewitt 2000). These two events may have resulted in the divergence of *Arborophila gingica* from *Arborophila rufogularis*, and that between several other species (Liu et al. 2012).

Our results suggest that the divergence between *Arborophila gingica guangxiensis* and *Arborophila gingica gingica* occurred 0.11 (0.05–0.19) mya, during or after the penultimate glaciation (0.13–0.42 mya). We speculate that *Arborophila gingica guangxiensis* and *Arborophila gingica gingica* might have had separate refugia during the glaciation, inducing population differentiation. This Pleistocene refugia scenario has been proposed for several bird species in southeast China, including *Tragopan caboti* (Dong et al. 2010) and *Alcippe morrisonia* (Song et al. 2009).

In any case, geographical isolation has likely played a role in population differentiation. *Arborophila gingica guangxiensis* and *Arborophila gingica gingica* are currently separated by the karst basin in central Guangxi. This area also represents unfavorable habitat for some montane species, including *Gorsachius magnificus* (Hu and Liu 2014) and *Stachyridopsis ruficeps* (Liu et al. 2012), perhaps due to the large portion of limestone in the karst basin. Early modern human activities dating back to 0.14 mya have been discovered in this area (Shen et al. 2002), and these activities might have interrupted gene flow between bird populations (Zhou and Jiang 2008). These isolation hypotheses may also have affected differentiation between *Arborophila gingica guangxiensis* and *Arborophila gingica gingica*.

The estimated temporal diversification and historical biogeography of *Arborophila gingica* proposed here is based on a small dataset and thus should ideally be substantiated with additional data. To better explore the underlying diversification process (e.g. speciation-with-gene-flow, Nosil 2008), more sampling using additional nuclear loci is needed (Edwards and Bensch 2009). In addition, ecological niche modelling may help to identify the potential distribution of both subspecies and the main environmental variables which determine the range of each subspecies (Hu and Liu 2014).

## Conclusion

Our study demonstrates that the newly found subspecies *Arborophila gingica guangxiensis* and nominate *Arborophila gingica gingica* formed reciprocal monophyletic groups in a multi-locus molecular phylogenetic analyses. The allopatric distribution of *Arborophila gingica guangxiensis* and *Arborophila gingica gingica* and a single diagnostic morphological difference underscore the distinctiveness of these two taxa (Zhou and Jiang 2008). The total body of evidence thus meets the traditional requirement that subspecies are geographically non-overlapping and phenotypically divergent (Wilson and Brown 1953; Mayr and Ashlock 1991) and meets the modern requirements that subspecies are either genetically distinct (Zink 2004), diagnosable (Remsen 2010) or both (Braby et al. 2012). Therefore, our results further support the taxonomic validity of *Arborophila gingica guangxiensis* and we suggest that this subspecies should be considered as a conservation unit.

## Supplementary materials

Supplementary material 1 Table S1 Data type: molecular data Explanation note: Primers used for PCR ampliﬁcation and sequencing in this study. This dataset is made available under the Open Database License (http://opendatacommons.org/licenses/odbl/1.0/). The Open Database License (ODbL) is a license agreement intended to allow users to freely share, modify, and use this Dataset while maintaining this same freedom for others, provided that the original source and author(s) are credited.De Chen, Qiong Liu, Jiang Chang, Aiwu Jiang, Fang Zhou, Yanyun Zhang, Zhengwang Zhang

Supplementary material 2 Figure S1 Data type: molecular data Explanation note: Phylogenetic trees from the nuDNA matrix and each of the nuclear introns. This dataset is made available under the Open Database License (http://opendatacommons.org/licenses/odbl/1.0/). The Open Database License (ODbL) is a license agreement intended to allow users to freely share, modify, and use this Dataset while maintaining this same freedom for others, provided that the original source and author(s) are credited.De Chen, Qiong Liu, Jiang Chang, Aiwu Jiang, Fang Zhou, Yanyun Zhang, Zhengwang Zhang
