# Supplementary material for: Multi-locus analysis supports the taxonomic validity of Arborophila gingica guangxiensis Fang Zhou & Aiwu Jiang, 2008
Source: Zookeys. 2016 Jan 20;(555):125–36. doi: 10.3897/zookeys.555.6814 (PMC4740825; doi:10.3897/zookeys.555.6814)
Supplement: Supplementary material 1 — Table S1 [file zookeys-555-125-s001.doc]

Table S1. Primers used for PCR ampliﬁcation and sequencing in this study.

| Locus and location | Primer sequence | Annealing T° | Source |
| --- | --- | --- | --- |
| **COI** | 5’-TTCTCCAACCACAAAGACATTGGCAC-3’ | 55° | Hebert et al. 2004 |
| Mitochondrion | 5’-ACGTGGGAGATAATTCCAAATCCTG-3’ |  |  |
| **CYTB** | 5’-TCAACCACACTTCACACAGGC-3’ | 50° | Chang et al. 2012 |
| Mitochondrion | 5’-GGTTTACAAGACCAATGTTTTTCA-3’ |  |  |
| **ND2** | 5’-CCCATACCCCRAAAATG-3’ | 54° | Sorenson et al. 1999 |
| Mitochondrion | 5’-CCTTATTTAAGGCTTTGAAGGC-3’ |  |  |
| **ALDOB** | 5’-GAGCCAGAAGTCTTACCTGAYGG-3’ | 58° | Kimball et al. 2009 |
| Z chromosome | 5’-CAGCTGTCACCATGTTNGG-3’ |  |  |
| **FGB** | 5’- CGCCATACAGAGTATACTGTGACAT-3’ | 54° | Marini and Hackett 2002 |
| 4th chromosome | 5’- GCCATCCTGGCGATTCTGAA-3’ |  |  |
| **G3PDH** | 5’-ACCTTTAATGCGGGTGCTGGCATTGC-3’ | 64° | Friesen et al. 1997 |
| 1st chromosome | 5’-CATCAAGTCCACAACACGGTTGCTGTA-3’ |  |  |
| **OVOG** | 5’-CAAGACATACGGCAACAARTG- 3’ | 52° | Armstrong et al. 2001 |
| 13th chromosome | 5’-GGCTTAAAGTGAGAGTCCCRTT-3’ |  |  |

Armstrong MH, Braun EL, Kimball RT (2001) Phylogenetic Utility of Avian Ovomucoid Intron G: A Comparison of Nuclear and Mitochondrial Phylogenies in Galliformes. Auk 118: 799–804.

Chang J, Chen D, Ye X, Li S, Liang W, Zhang Z, Li M (2012) Coupling Genetic and Species Distribution Models to Examine the Response of the Hainan Partridge (*Arborophila ardens*) to Late Quaternary Climate. PLoS ONE 7: e50286.

Friesen VL, Congdon BC, Walsh HE, Birt TP (1997) Intron variation in marbled murrelets detected using analyses of single-stranded conformational polymorphisms. Molecular Ecology 6: 1047–1058.

Hebert PDN, Stoeckle MY, Zemlak TS, Francis CM (2004) Identification of Birds through DNA Barcodes. PLoS Biology 2: e312.

Kimball RT, Braun EL, Barker FK, Bowie RC, Braun MJ, Chojnowski JL, Hackett SJ, Han K-L, Harshman J, Heimer-Torres V (2009) A well-tested set of primers to amplify regions spread across the avian genome. Molecular Phylogenetics and Evolution 50: 654–660.

Marini MÂ, Hackett SJ (2002) A Multifaceted Approach to the Characterization of an Intergeneric Hybrid Manakin (Pipridae) from Brazil. Auk 119: 1114–1120.

Sorenson MD, Ast JC, Dimcheff DE, Yuri T, Mindell DP (1999) Primers for a PCR-Based Approach to Mitochondrial Genome Sequencing in Birds and Other Vertebrates. Molecular Phylogenetics and Evolution 12: 105–114.
