# Supplementary figures and images for: Multi-locus analysis supports the taxonomic validity of Arborophila gingica guangxiensis Fang Zhou & Aiwu Jiang, 2008
Source: Zookeys. 2016 Jan 20;(555):125–36. doi: 10.3897/zookeys.555.6814 (PMC4740825; doi:10.3897/zookeys.555.6814)

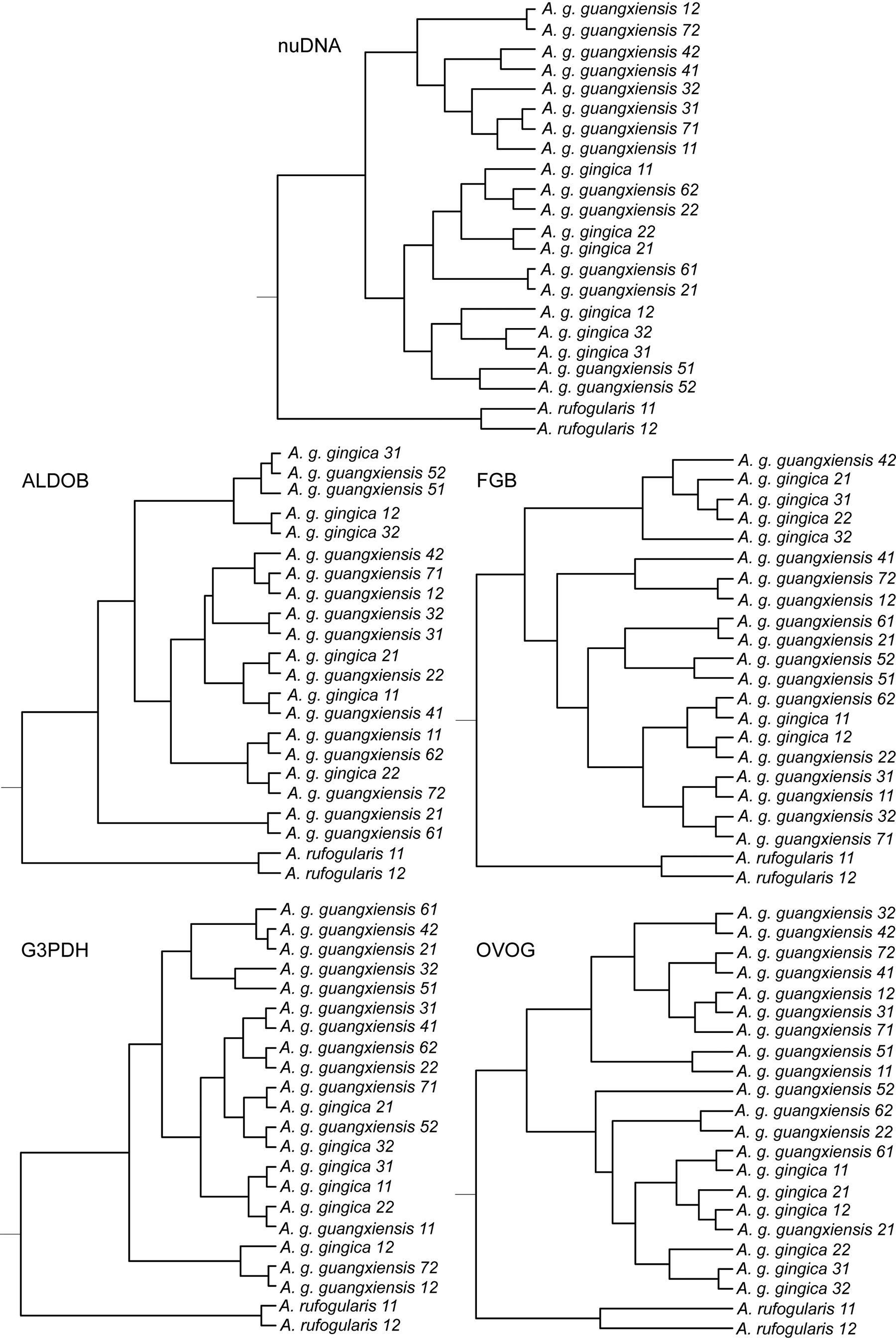

Supplement: Supplementary material 2 — Figure S1 [file zookeys-555-125-s002.tif]
